# A Comparison of Surrogate Behavioral Models for Power Amplifier Linearization under High Sparse Data

**DOI:** 10.3390/s22197461

**Published:** 2022-10-01

**Authors:** Jose Alejandro Galaviz-Aguilar, Cesar Vargas-Rosales, José Ricardo Cárdenas-Valdez, Daniel Santiago Aguila-Torres, Leonardo Flores-Hernández

**Affiliations:** 1 Tecnologico de Monterrey, School of Engineering and Sciences, Monterrey 64849, Mexico; 2 IT de Tijuana, Tecnológico Nacional de México, Tijuana 22435, Mexico

**Keywords:** behavioral modeling, linearization model, machine learning, nonlinear modeling, power amplifier

## Abstract

A good approximation to power amplifier (PA) behavioral modeling requires precise baseband models to mitigate nonlinearities. Since digital predistortion (DPD) is used to provide the PA linearization, a framework is necessary to validate the modeling figures of merit support under signal conditioning and transmission restrictions. A field-programmable gate array (FPGA)-based testbed is developed to measure the wide-band PA behavior using a single-carrier 64-quadrature amplitude modulation (QAM) multiplexed by orthogonal frequency-division multiplexing (OFDM) based on long-term evolution (LTE) as a stimulus, with different bandwidths signals. In the search to provide a heuristic target approach modeling, this paper introduces a feature extraction concept to find an appropriate complexity solution considering the high sparse data issue in amplitude to amplitude (AM-AM) and amplitude to phase AM-PM models extraction, whose penalties are associated with overfitting and hardware complexity in resulting functions. Thus, experimental results highlight the model performance for a high sparse data regime and are compared with a regression tree (RT), random forest (RF), and cubic-spline (CS) model accuracy capabilities for the signal conditioning to show a reliable validation, low-complexity, according to the peak-to-average power ratio (PAPR), complementary cumulative distribution function (CCDF), coefficients extraction, normalized mean square error (NMSE), and execution time figures of merit. The presented models provide a comparison with original data that aid to compare the dimension and robustness for each surrogate model where (i) machine learning (ML)-based and (ii) CS interpolate-based where high sparse data are present, NMSE between the CS interpolated based are also compared to demonstrate the efficacy in the prediction methods with lower convergence times and complexities.

## 1. Introduction

Presently, given the continuous growth and development of modern wireless communication technologies, DPD is widely used for the linearization of power amplifiers. However, the introduction of base stations (i.e., femtocells, picocells, and microcells) limits the DPD hardware complexity and power consumption and requires faster adaption because the PA’s behavior may vary more often, which makes high-complexity methods unsuitable. In addition, Radio-frequency/Microwave transmission circuits with wide dynamic bandwidths represent a challenge for modeling and DPD linearization techniques. Behavioral modeling based on higher-order polynomial systems for long-term and short-term induced memory represents an efficient methodology for improving spectral efficiency [1,2]. The selection of the primary method for the modeling and linearization stages is essential if it is required to use multivariable data, gain precision, and not increase the involved coefficients during hardware implementation [3]. Various efforts to improve accuracy and reduce coefficients have been developed as alternative techniques for out-of-band emission control. In this work, a training was performed to extract the inverse characteristics in terms of the normalized output using a small-signal gain of the PA-Tx system. In [4], a PAPR reduction is reached based on orthogonal frequency division multiplexing (OFDM); in this case, the error vector magnitude (EVM) for multiple-input multiple-output (MIMO) is evaluated as a metric. The developed system comprises a MIMO precoder, resource mapper, OFDM-PAPR reduction stage, and OFDM modulation scheme processed before the PA. In [5], a parallel structure of two boxes is developed for the modeling and DPD stage, the coefficient extraction method is based on applying an indirect learning structure and a least squares (LS) method; the main proposal is based on coefficient reduction up to 22% for a signal with 64-QAM. An example of feature selection techniques for DPD linearization with order reduction methods is presented as an alternative to reduce the high volume of coefficients of the Volterra Series [6], this work is applied to improve the predistortion performance for a class J-PA using a dual-band scenario. Consequently, in [7] spline-interpolated LUT methods are proposed for baseband applications built on special truncations of the Volterra Series such as Wiener, Hammerstein, and a combination of both; while the observation path uses a general scheme based on an indirect learning approach (ILA) structure for LTE-based systems 5G-new radio (NR). Despite existing works, the need for systems with accurate data analysis, ability to perform automated modeling/linearization processes, and big data management is still present, and in this regard, machine learning (ML) represents an efficient proposal. Artificial neural networks have been used in over-the-air (OTA) linearization for a highly nonlinear and wide-band mmWave active phased array (APA) transmitter, and results indicate that they are capable of learning distinctive nonlinear distortions, which may not easily fit to existing polynomial-based solutions [8].

In [9], an extreme learning approach is developed for the modeling stage compared to network learning algorithms in conventional neural networks, and it is applied to a Class-E PA. To balance computational efficiency and accuracy of the modeling stage, a low-complexity wireless method called simplified generalized memory polynomial, and a random forest process for classification are proposed [10]. In [11], a random forest application for DPD based on Volterra is developed; this approach is validated for a PA under 5G-NR generation signal, improving the coefficient extraction methodology. The divide-and-conquer paradigm can offer a solution while using calculation of multiple small-scale LS solutions which is easier and less complex than that of a large-scale LS problem. For addressing the latter case, probabilistic models that allow different submodels were proposed [12,13,14] to cover both PA modeling and DPD linearization with a double objective using a mixture of expert techniques for wide-band signals.

In this work, three model structures are presented to demonstrate rigorous approximations that can aid in the search for adaptive algorithms and are addressed in order to find specific functions that have been acquired from a training process either for a supervised or unsupervised learning conducted with a data-driven approach based on input and output samples. Thus, the prediction characteristics are explored for classification goals on different sparse feature data in order to extract and provide a decision model based on a feature inspection to provide an appropriate PA and /or PD model concerning this problem in a regression fashion technique considering two ML approaches. An application for determining digital predistorter model structures for wideband power amplifiers for 5G-NR signals through RF is proposed in [11]. Among different ML techniques, Decision Trees remain as one of the simplest learning approaches due to the method producing a comprehensive model from a given dataset [15]. More accurate results are obtainable with Decision Trees on nonlinear problems and tend to be mildly less accurate on problems with a linear structure [16]. RF and decision tree algorithms are widely popular for many prediction applications and are characterized by having few tuning parameters and working with high-dimensional data.

Sparse systems are a recurrent phenomenon that occurs while acquiring PA measurement curves for systems that operate with MIMO technologies and applications related to the new 5G standard [17]. In recent research [18], an extension of a sparse-Bayesian treatment is developed to remove non-active regressors and re-estimate the model coefficient in a system with varying power levels. Additionally, in [19], a dynamic deflection reduction algorithm was developed due to the high performance of beneficial terms and reduced schemes compared to the substantial full Volterra model. However, alternative approaches, such as those derived from ML with a considerable reduction of coefficients, are possible. In this article, we extend our preliminary work in [20], where the basic ML modeling was considered for direct modeling of PAs compared against complex spline-interpolating models (b-splines and cubic-splines) in terms of explicit complexity, modeling accuracy, and linearization performance. Therefore, this paper is concentrated particularly in the search of linearization capabilities of the proposed models, where the high sparse data in the PA characteristics (AM-AM and AM-PM responses) yields to a substantial increase in the processing complexity.

The remainder of this paper is organized as follows: In Section 2, the theory and its extension to the modeling is presented to different basis structures and its associated algorithms. Section 3 describes the experimental setup and illustrates the proposed test and training setup for ML models with different bandwidths for 2.45 GHz band applications. Section 4 shows the developed RT, RF, and the cubic-spline algorithm for approximate data modeling and discusses the FPGA implementation complexity. Finally, Section 5 depicts the obtained conclusions.

## 2. Modeling Framework

**Figure 1:**
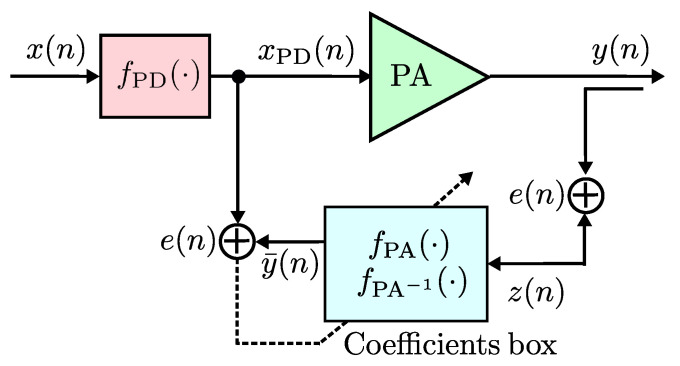
General diagram of the proposed behavioral model on indirect learning approach.

We introduce three model validations that are used to provide an algorithm construction, used to perform the PA modeling and obtain the PD linealization model. In the context of the algorithm construction to pursue modeling capabilities, a system is built based on an indirect learning architecture as shown in Figure 1.

**Figure 2:**
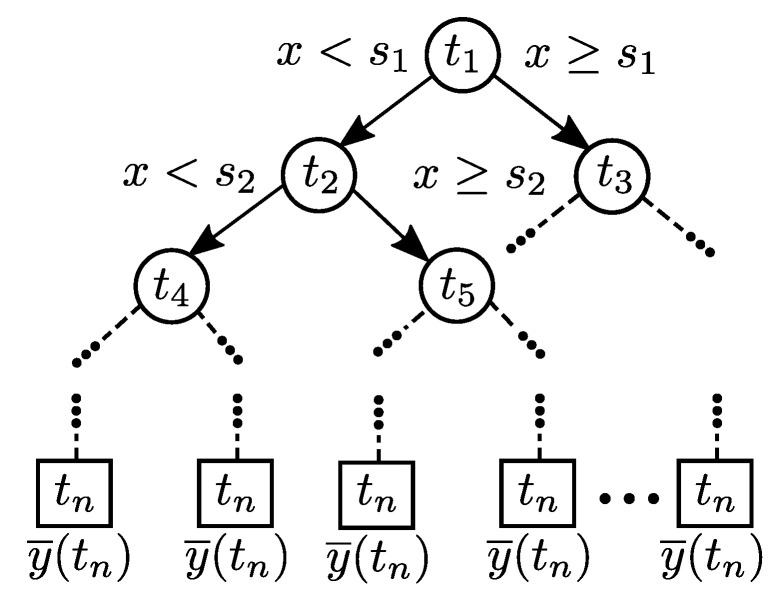
General diagram of a RT.

Following these ideas, initially in this work an ensemble approach is presented to obtain the PA behavioral modeling with the Classification and Regression Trees (CART) supervised learning algorithm. This method performs repeated splits on a training dataset until a rule establishes a terminal condition. Each split becomes a node of a tree and terminal nodes contain the response of the modeling system [16]. Decision Trees are conceptually simple, have good enough learning performance, and are computationally cheap in comparison with other learning methods, with training routines available in a wide range of programming languages. Because of their rule-based nature, tree-based methods are also appealing in terms of interpretability which makes them popular in many applications [21]. Commonly, a good approximation is obtained to highly nonlinear models using a cost function. Figure 2 shows a general diagram of a RT.

Making a prediction requires traversing the tree from the root node to a leaf node. Since Decision Trees are approximately balanced, traversing through the tree to make a prediction requires roughly $\log_{2}\left(n\right)$ nodes, where *n* is the number of training samples. For simplicity the model can be constructed with each sample of the PA input signal $x_{n}$ which is transformed into a feature vector $X_{n}$. The vector $X_{n}$ in Equation (1) serves as the input of the model.

(1) $$X_{n}=\left\{z_{n},z_{n-1},\ldots ,z_{n-m}\right\}\in R^{3m}$$

where $z_{n}$ is a vector containing the real, imaginary, and absolute values of the PA input signal, and *m* is a memory parameter which includes lagged samples of the input signal. The $z_{n}$ vector is described as follows

(2) $$z_{n}=\{ℜ\left(x_{n}\right),ℑ\left(x_{n}\right),|x_{n}\left|\right\}\in R^{3}$$

The model output $\bar{y}$ is then computed in an ensemble fashion with a set of regression trees RTs, which is defined in Equation (3). Since standard CART is limited to real valued inputs and outputs, different models are trained to produce the complex output.

(3) $$\bar{y}=y_{1}\left(X\right)+y_{2}\left(X\right)i+\epsilon_{1}\left(X\right)+\epsilon_{2}\left(X\right)i$$

having the complex signal I and Q components as a set of RT $y_{1}$ and $y_{2}$, and an estimated error term $\epsilon_{1}$ and $\epsilon_{2}$ trained on the difference between $y_{1}$, $y_{2}$ and the PA output signal *y*. The estimated error terms are given as

(4) $$\begin{array}{cc} \; & \epsilon_{1}\left(X\right)=y_{1}\left(X\right)-ℜ\left(y\right) \end{array}$$

(5) $$\begin{array}{cc} \; & \epsilon_{2}\left(X\right)=y_{2}\left(X\right)-ℑ\left(y\right) \end{array}$$

The calculated value $\bar{y}$ of Equation (3) is the complex output of the model. The estimated error term is used to further increase prediction accuracy.

### 2.1. Model Identification with Regression Tree

**Figure 3:**
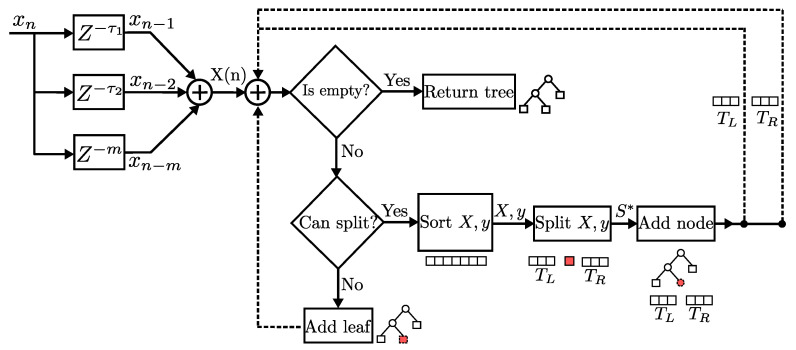
Block diagram of the construction process of a RT.

RTs are a model-based supervised learning technique which requires building a tree out of examples in a training dataset, in order to use that model to make predictions. The construction process of a RT is depicted in Figure 3 as a block diagram. The model identification stage starts by obtaining a vector of delayed values of the PA input signal $x_{n}$. The values of the vector are compared, if data are available, then the terminal condition is tested. For non-terminal nodes, the values are sorted and a split is computed, this results in a left and right subset of the vector which are used as new inputs to the process. As terminal nodes are added to the tree, the construction process continues recursively until no more data are available.

The process of constructing a Decision Tree using the CART algorithm can be estimated using computational complexity theory. In this kind of analysis, the time and space resources required to run an algorithm are calculated for any input of size *n*. Big O notation is used as a mathematical description of the growth of a function, this approach is useful due to measurement inconsistencies that arise given different algorithm parameterization, initialization, hardware implementation, data representation, and other factors. Decision Trees follow a divide and conquer scheme with runtime of $O(mn\log_{2}n)$ where *n* is the number of samples and *m* is the number of features. However, since the algorithm grows the tree recursively and repeats the process many times, the runtime can exponentially scale up to $O(m^{k}n^{q})$ where *k* and *q* can be any constant c≤2 [15]. In this particular case, the number of features corresponds to the memory depth assigned to the modeling system. We consider two cases to denote the modeling complexity:

Case 1: In the interest of validating the complexity estimation of the algorithm, a datasetof a previous work is used to evaluate resource use. The dataset was collected from a GaN HEMT Class-AB PA with carrier frequency 2.0-GHz using an LTE-A 10-MHz bandwidth signal comprising 65,531 samples [20].

Case 2: Although this work focuses on a dataset based on a ZX60-V63+ PA which uses a carrier frequency 2.45 GHz and LTE 10-MHz and LTE 15-MHz signals with 30,720 samples, the bigger dataset is deliberately chosen to extrapolate on the algorithms resource use for greater sample sizes. The convergence running times are performed on an Intel Core i7-4790 CPU @ 3.60 GHz machine running MATLAB 2019b.

**Figure 4:**
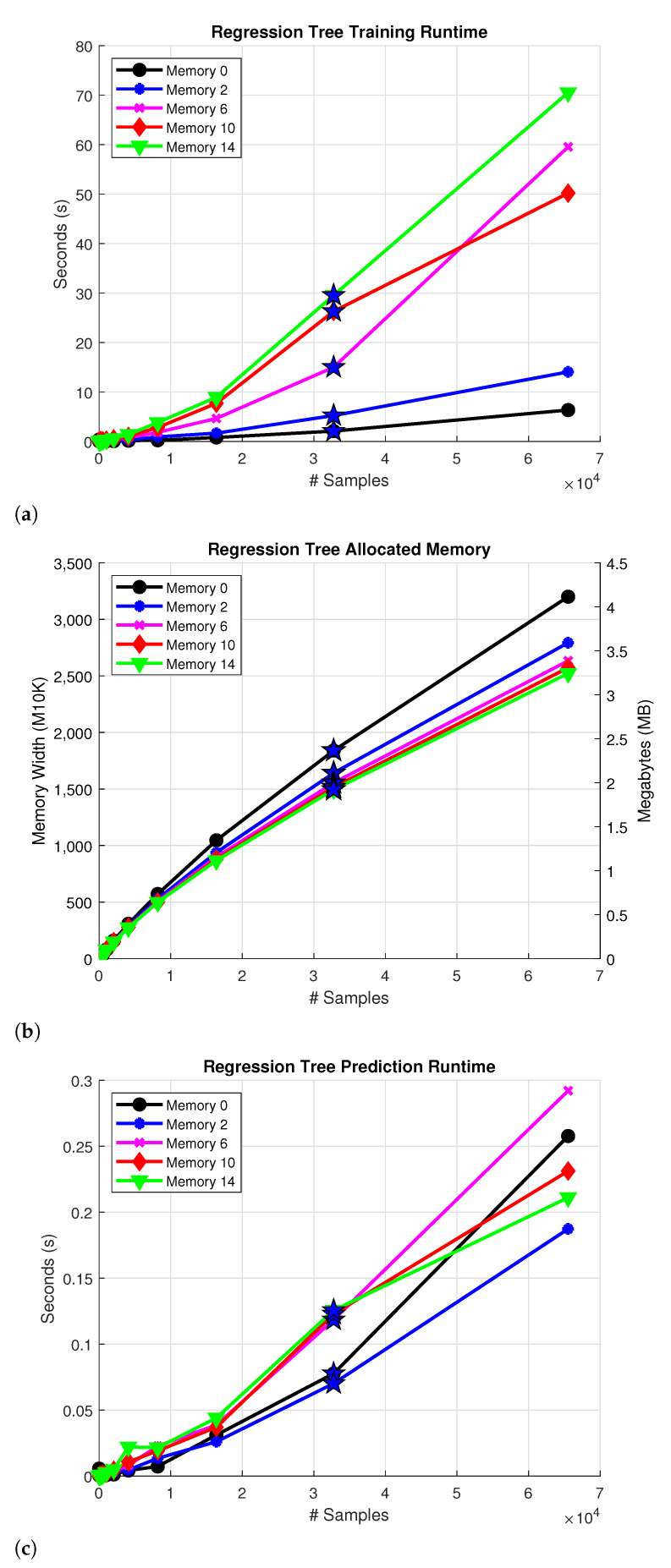
Complexity for the; (**a**) Construction process of a RT and (**b**) Allocated memory of the RT model (**c**) Prediction runtime of the model. The star denotes the Case 2 approximation results.

The algorithm is implemented using the MATLAB function fitrtree that trains the model depending on different parameters. The MinParentSize and MinLeafSize parameters are used as the stopping criteria, the former refers to the minimum number of observations for each branch in the tree and the latter refers to the least number of observations per leaf. Both of these parameters are set to 1. The MergeLeaves and Prune parameters are set to off in order to speed up tree construction. Figure 4a portrays measurements of the training process runtime using different input sample sizes and memory values. The measured execution time of the algorithm increases with the number of input samples and memory depth according to the estimated complexity $O(mn\log_{2}n)$. A star marker is used to denote the results of the Case 2 experiments, which are the main focus of these comparisons.

The model sizes are calculated in bytes of memory as a reference for comparison. For this, the default data structures are changed from double to single precision floating point numbers to optimize memory resources. Despite running a simulated framework on MATLAB, an FPGA hardware resource estimation is made by calculating the number of M10K memory blocks required to store the model using the conversion 1 block equal to 10 kilobits. Figure 4b outlines the allocated memory for each instance with a single floating point representation of the values. The memory usage increases as the number of input samples becomes greater, but the same does not necessarily apply for the memory parameter. This is explained on the grounds that the algorithm selects the best split based on the input features, in this case memory, and depending on that some trees end up with more nodes than others. For example, when analyzing the model with memory 0, the depths of trees $y_{1}$, $y_{2}$, $\epsilon_{1}$, and $\epsilon_{2}$ are 44, 37, 397, and 419. In contrast, the trees $y_{1}$, $y_{2}$, $\epsilon_{1}$, and $\epsilon_{2}$ of the model with memory 14 are 35, 37, 196, and 11.

The complexity of the prediction runtime mainly depends on the depth of the tree, and it is significantly smaller in comparison to the training procedure runtime. In order to accommodate the data structures changes from double to single precision floating point numbers, a custom C implementation of the predict procedure is used. Figure 4c presents the measured runtime of the algorithm in seconds for different sample sizes and memory values. Since making a prediction requires traversing the tree, and RTs are approximately balanced, the prediction process has an estimated complexity of $O(\log_{2}\left(n\right))$, where *n* is the sample size. It is noted that the memory value does not appear in the complexity expression, this is why memory does not significantly impact the prediction runtime as observed in Figure 4c.

### 2.2. Regression Tree Framework

Algorithm 1 describes the training procedure of the model, where a RT is constructed from a training dataset in a recursive manner. The algorithm tests whether or not the training dataset can be split into two subsets, if that is the case, then it computes the mean squared error (MSE) of the response values and their corresponding mean. At this point, the input and response values are sorted for each feature in the training dataset and different splits are proposed. The splits are evaluated by calculating the MSE of the left and right subsets, and the split that maximizes the MSE decrease is chosen for that node in the tree. This process is then repeated for the left and right subsets of the tree until the terminal condition is met. The MSE is defined with $y_{i}$ as the observed value and $\bar{y_{i}}$ as the corresponding predicted value as follows

(6) $$\mathrm{MSE}=\frac{1}{n}\sum_{i}^{N}{\left(y_{i}-\bar{y_{i}}\right)}^{2}$$

The model trains four RTs in order to make predictions. The first tree estimates the I component of the output signal, likewise the second tree outputs the Q component of the output signal. These two trees are evaluated and an error of the model is estimated. A second set of trees is trained to predict the estimated error. To summarize, this method is described by the following steps:

1. Obtain the input vector *X* which contains *m* delays of the input signal *x*.
2. Construct the RTs $y_{1}$ and $y_{2}$ for the real and imaginary components of the signal.
3. Evaluate $y_{1}$ and $y_{2}$ and compute the difference between their prediction and the PA output signal.
4. Construct the RTs $\epsilon_{1}$ and $\epsilon_{2}$ for the real and imaginary components of the estimated error.
5. Compute $\bar{y}$ using Equation (3).

**Algorithm 1** Training procedure for Regression Tree.  1:**procedure** TRAIN_RT_TREE(*T*)  2:    **if** CAN_SPLIT(T) **then**  3:        $X_{T},y_{T}\leftarrow \;T$  4:        $\epsilon_{t}\leftarrow \frac{1}{n}\sum_{t\in T}{\left(y_{t}-\mathrm{MEAN}\left(y_{T}\right)\right)}^{2}$  5:        **for** $f\in X_{t}$ **do**  6:           $\mathrm{sort}\;X_{T},y_{T}\mathrm{in}\mathrm{ascending}\mathrm{order}\mathrm{by}X_{T,f}$  7:           $s^{*}\leftarrow \mathrm{NULL}$  8:           **for** s∈S **do**  9:               $T_{L},T_{R}\leftarrow s$10:               $\epsilon_{t_{L}}\leftarrow \frac{1}{n}\sum_{t\in T_{L}}{\left(y_{t}-\mathrm{MEAN}\left(y_{T_{L}}\right)\right)}^{2}$11:               $\epsilon_{t_{R}}\leftarrow \frac{1}{n}\sum_{t\in T_{R}}{\left(y_{t}-\mathrm{MEAN}\left(y_{T_{R}}\right)\right)}^{2}$12:               $\Delta \epsilon \leftarrow \epsilon_{t}-\epsilon_{t_{L}}-\epsilon_{t_{R}}$13:               $s^{*}\leftarrow \mathrm{MAX}\left(\Delta \epsilon \right)$14:           **end for**15:        **end for**16:        $t_{L}\leftarrow \mathrm{TRAIN}\_\mathrm{RT}\_\mathrm{TREE}\left(T_{L}\right)$17:        $t_{R}\leftarrow \mathrm{TRAIN}\_\mathrm{RT}\_\mathrm{TREE}\left(T_{R}\right)$18:        **return** $X_{t,f},y_{t},t_{L},t_{R}$19:    **else**20:        **return** NULL,MEAN(y),NULL,NULL21:    **end if**22:**end procedure**23: 24:**procedure** TRAIN_RT(T)25:    X,y←T26:    $y_{1}\leftarrow \mathrm{TRAIN}\_\mathrm{RT}\_\mathrm{TREE}\left(X,ℜ\left(y\right)\right)$27:    $y_{2}\leftarrow \mathrm{TRAIN}\_\mathrm{RT}\_\mathrm{TREE}\left(X,ℑ\left(y\right)\right)$28:    $\bar{y}\leftarrow y_{1}\left(X\right)+y_{2}\left(X\right)i$29:    $\bar{\epsilon }\leftarrow y-\bar{y}$30:    $\epsilon_{1}\leftarrow \mathrm{TRAIN}\_\mathrm{RT}\_\mathrm{TREE}\left(X,ℜ\left(\bar{\epsilon }\right)\right)$31:    $\epsilon_{2}\leftarrow \mathrm{TRAIN}\_\mathrm{RT}\_\mathrm{TREE}\left(X,ℑ\left(\bar{\epsilon }\right)\right)$32:    **return** $y_{1},y_{2},\epsilon_{1},\epsilon_{2}$33:**end procedure**

Notation: *T*, $T_{L}$, $T_{R}$, denote the training dataset, the left subset of a split, and the right subset of a split. The input and response values $X_{t,f}$ and $y_{t}$ are contained within *T*. The *S* values correspond to the proposed splits and $\epsilon_{t}$, $\epsilon_{t_{L}}$, $\epsilon_{t_{R}}$, Δϵ represents the MSE of the response values, the MSE of the left subset of a split, the MSE of the right subset of a split, and the MSE decrease of a split. The $y_{1}$, $y_{2}$, $\epsilon_{1}$, and $\epsilon_{2}$ are the RTs obtained after the training procedure.

### 2.3. Random Forest Framework

RF is a supervised learning algorithm for general purpose classification and regression tasks. RF consist of a collection of Decision Trees, where each tree is constructed using randomly selected subsets of predictor variables, and the output is computed by aggregating the predictions for each observation [22]. The method is recognized for its accuracy and ability to work on small sample sizes and high-dimensional feature spaces [23]. The algorithm grows *M* randomized RTs. Before the construction of each tree, $a_{n}$ observations are randomly sampled from the original dataset and these observations are the only ones used in the construction process for that tree. The output value at input *x* is denoted by $m_{n}\left(x,\Theta_{j},D_{n}\right)$, having $D_{n}$ as the original dataset and $\Theta_{1},\ldots ,\Theta_{M}$ as an independent random variable which is used to resample the original dataset prior to training. The trees are combined to form the prediction as Equation (7).

(7) $$m_{M,n}\left(x,\Theta_{1},\ldots ,\Theta_{m},D_{n}\right)=\frac{1}{M}\sum_{j=1}^{M}m_{n}\left(x;\Theta_{j},D_{n}\right)$$

**Figure 5:**
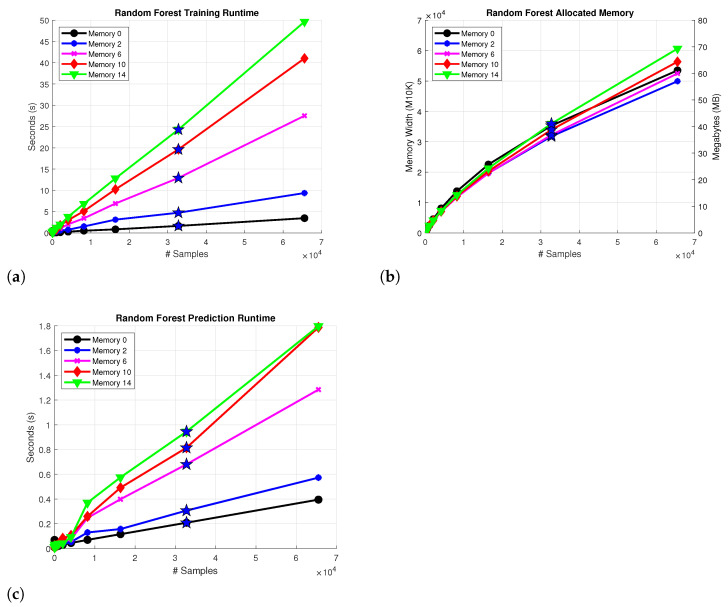
Complexity for the; (**a**) Construction process of a RF and (**b**) Allocated memory of the RF model (**c**) Prediction runtime of the model. The star denotes the Case 2 approximation results.

The RF training procedure is described in Algorithm 2. The procedure trains *M* different trees using randomly sampled subsets $a_{i}$ of the dataset and also a randomly sampled subset of input features M. In this particular case, M refers to the memory values included as inputs. For PA modeling, a model has to be constructed for the real and imaginary values of the complex output signal, in a similar fashion to RTs. Since RF uses a combination of trees, the time and space complexity of the algorithm also depends on the construction process of the aforementioned trees. The MATLAB TreeBagger class is used as the implementation of RF, with the MinLeafSize parameter set to 1 and the MinParentSize parameter set to 10 as the stopping criteria. The total number of trees *M* is set to 12; therefore, an increase in resource usage is expected as a result of using six times as many trees in comparison to the RT approach. Figure 5 depicts (a) the training runtime for the RF algorithm with different memory values (b) the allocated memory for each instance with a double floating point representation of the values (c) the prediction runtime of the model. The training procedure executes faster than RT due to the different parametrization on MinParentSize, which reduces the tree size and consequently the number of iterations that are performed during training. The expected 6 times increase is reflected on the prediction runtime; however, the memory consumption of the algorithm is aggravated considering the use of more trees with double floating point representations instead of single floating point, which in turn results in more allocated memory.
**Algorithm 2** Training procedure for Random Forest.  1:**procedure**TRAIN_RF_TREE(*T*)  2:    **if** CAN_SPLIT(T) **then**  3:        $X_{T},y_{T}\leftarrow \;T$  4:        $\epsilon_{t}\leftarrow \frac{1}{n}\sum_{t\in T}{\left(y_{t}-\mathrm{MEAN}\left(y_{T}\right)\right)}^{2}$  5:        $\mathrm{Sample}\mathrm{subset}M\mathrm{uniformly}\mathrm{in}X_{t}$  6:        **for** f∈M **do**  7:           $\mathrm{sort}\;X_{T},y_{T}\mathrm{in}\mathrm{ascending}\mathrm{order}\mathrm{by}X_{T,f}$  8:           $s^{*}\leftarrow \mathrm{NULL}$  9:           **for** s∈S **do**10:               $T_{L},T_{R}\leftarrow s$11:               $\epsilon_{t_{L}}\leftarrow \frac{1}{n}\sum_{t\in T_{L}}{\left(y_{t}-\mathrm{MEAN}\left(y_{T_{L}}\right)\right)}^{2}$12:               $\epsilon_{t_{R}}\leftarrow \frac{1}{n}\sum_{t\in T_{R}}{\left(y_{t}-\mathrm{MEAN}\left(y_{T_{R}}\right)\right)}^{2}$13:               $\Delta \epsilon \leftarrow \epsilon_{t}-\epsilon_{t_{L}}-\epsilon_{t_{R}}$14:               $s^{*}\leftarrow \mathrm{MAX}\left(\Delta \epsilon \right)$15:           **end for**16:        **end for**17:        $t_{L}\leftarrow \mathrm{TRAIN}\_\mathrm{RF}\_\mathrm{TREE}\left(T_{L}\right)$18:        $t_{R}\leftarrow \mathrm{TRAIN}\_\mathrm{RF}\_\mathrm{TREE}\left(T_{R}\right)$19:        **return** $X_{t,f},y_{t},t_{L},t_{R}$20:    **else**21:        **return** NULL,MEAN(y),NULL,NULL22:    **end if**23:**end procedure**24: 25:**procedure**TRAIN_RF(T,M)26:    $D_{n}\leftarrow T$27:    **for** m∈M **do**28:        $\mathrm{Sample}\mathrm{points}a_{i}\mathrm{uniformly}\mathrm{in}D_{n}$29:        $m\leftarrow \mathrm{TRAIN}\_\mathrm{RF}\_\mathrm{TREE}(a_{i})$30:    **end for**31:    **return** *M*32:**end procedure**

### 2.4. Surrogate Cubic-Spline Precomputed Model Approach

**Figure 6:**
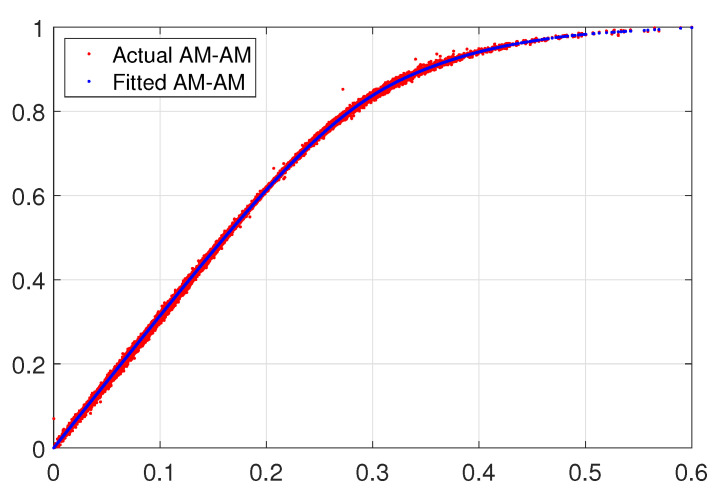
Approximation for a fitted model with polynomial basis.

**Figure 7:**
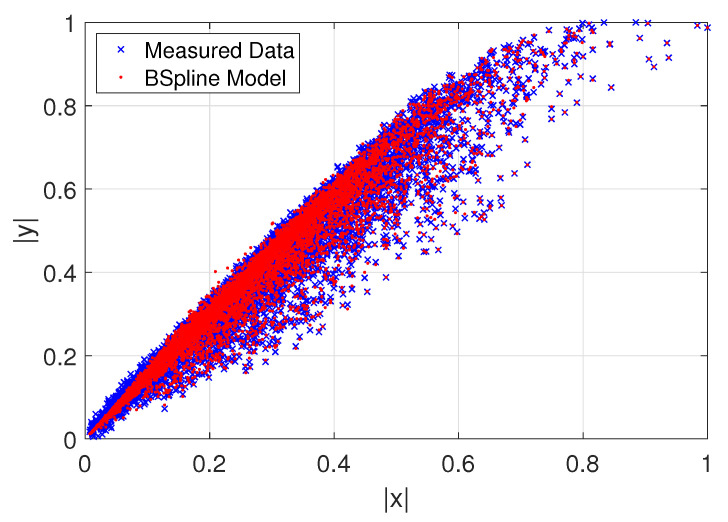
Approximation model with b-spline basis.

For the first model capabilities demonstration of the CS, an AM-AM behavioral curve whose thickness with very smooth data can be extracted with a fast convergence speed with lower training samples for a polynomial complexity given as shown in Figure 6, while in Figure 7 we exemplify an AM-AM behavior that shows a thick high sparse data that increase the extraction complexity. Therefore, we formulate the mathematical models with the Bézier polynomial curves that can be described in terms of splines y(u) using also the combination of allied to control points $c_{i}$, as described in the standard Formula (8), unconditional stability is guaranteed. Additionally, $N_{i}^{n}\left(u\right)$ are piece-wise polynomial functions with finite support that satisfies certain continuity conditions. In the recurrence fashion relationship to define b-splines basis, it is considered for simplicity a sequence ($a_{i}$) doubly infinite of simple knots, such that $a_{i}<a_{i+1}$ for all *i*. Therefore, the b-splines $N_{i}^{n}$ are defined through the recurrence relationship as in (9). In the base model we use two parameters that are determined to not only maintain stability but also ensure the desired accuracy. Thus, this model is a simple step-by-step algorithm denoted by (8). Therefore, the stability and accuracy analysis of the b-spline algorithm exhibits an efficiency and computational cost viable for the proposed method and is demonstrated through two numerical simulations [24].

(8) $$\begin{array}{cc} y(u) & =\sum c_{i}N_{i}^{n}\left(u\right) \end{array}$$

(9) $$\begin{array}{cc} N_{i}^{0}\left(u\right) & =\left\{\begin{array}{cc} 1 & \mathrm{if}\;\;u\;\in [a_{1},a_{1}+1)\\ 0 & \mathrm{otherwise} \end{array}\right.\\ \; & N_{i}^{0}=\alpha_{i}^{n-1}N_{i}^{n-1}\left(u\right)+\left(1-\alpha_{i+1}^{n-1}\right)N{i+1}^{n-1}\left(u\right) \end{array}$$

where

(10) $$\begin{array}{cc} \; & \alpha_{i}^{n-1}=\left(u-a_{i}\right)/\left(a_{i+n}-a_{i}\right) \end{array}$$

is a local parameter with respect to the support of $N_{i}^{n-1}$. Thus, for b-spline recursive as in (9), it is found by iterative determining the most relevant functions according to the reduction linearization coefficients, the model can be simply derived as a cubic-spline basis function in terms of gain expansion $G_{m}$ described as

(11) $$\begin{array}{cc} y(n) & =\sum_{m=0}^{ML-1}G_{m}^{}{}_{\;}\;\;{\left|z\left(n-m\right)\right|}^{2}z\left(n-m\right) \end{array}$$

where y(n) denotes the received RF signal associated to an *m* memory depth value. Following the notation in a recursive way, the containing gain factors $G_{m}\left(x\right)$ are expanded in terms of 1D cubic-spline basis functions given by,

(12) $$G_{m}\left(x\right)=\sum_{j=1}^{J}a_{j}B_{j}\left(x\right)$$

where $a_{j}$ corresponds to the real-valued coefficients of the *j*-th spline expansion, and the $B_{j}\left(x\right)$ function. LS solution is used to develop an error model with the goal of minimizing estimations, with a matrix definition given as,

(13) $$\begin{array}{cc} G_{m}\left(x\right) & =\sum_{i=0}^{N_{k}-1}c_{i}\left(m\right)\phi_{i}{\left(|x\right|}^{2})\\ & \phi_{i}{\left(|x\right|}^{2})≜\;\delta_{i,u}\\ & =\left\{\begin{array}{cc} 1 & \forall \;i=u\\ 0 & \forall \;\;\mathrm{other}\;\mathrm{knots} \end{array}\right.\\ & \forall \;i\;\in \;\left[0,N_{k}\right],\forall \;u\;\in \;\left[0,N_{k}-1\right] \end{array}$$

where $c_{i}\left(m\right)$ are the weight model coefficients and the gain function $G_{m}\left(x\right)$ for each memory delay index *m*, and $N_{k}$ number of splines. In (13) the uniform $\phi_{i}$ basis functions with an entire amplitude span decay swiftly from 1 at (u) to 0 at all other knots thus yield to evaluate the limits of a memoryless PA and deterministic memory mitigation. Thus, a smooth function degree $N_{k}$, as the value and first $N_{k-1}$ derivatives of the spline function will be continuous [25]. The one-dimensional cubic B-splines $\left(N_{k}=3\right)$ are for instance the most common form, and have continuous value, first, and second derivatives.

## 3. Implementation and Measurement

### Experimental Setup Testbed

The captured signals are then used for training and testing of different ML models. The PA testbed can perform signal playback and data capture from the PA device under test assuming different instantaneous bandwidths (IBWs). The experimental setup considers the acquisition of real-valued data from several run tests to calculate crest factor reduction (CFR), PAPR, and adjacent channel power ratio (ACPR) signal measurements considering the device under test (DUT).

Therefore, in the proposed system, a transceiver software-defined radio (SDR) base makes suitable the model’s implementation and the experimental results. The SDR platform consists of a system-on-chip (SoC) device to provide a software and hardware interface to develop an SDR system. Moreover, the transceiver board uses an interface (AXI) interconnect which provides very low latency with high throughput. The radio-frequency conversion system includes a direct conversion with 2×2 channels for the *(I)* in-phase and *(Q)* quadrature signals. The system also includes a filter stage with an interpolation 128-tap type finite impulse response (FIR) that can handle and control by an adjustable internal sampling rate to produce a 12-bit output signal. The testbed setup considers a ZX60-V63+ power amplifier DUT with 50 ohms output impedance, biased with a 5 V bias, and provides a typical gain of around 20 dB under a 2.45-GHz frequency operating condition. In [12], a DPD linearization is validated for LTE and 256-QAM modulated signals that can linearize the dynamic nonlinear of the ZX60-V63+ PA.

**Figure 8:**
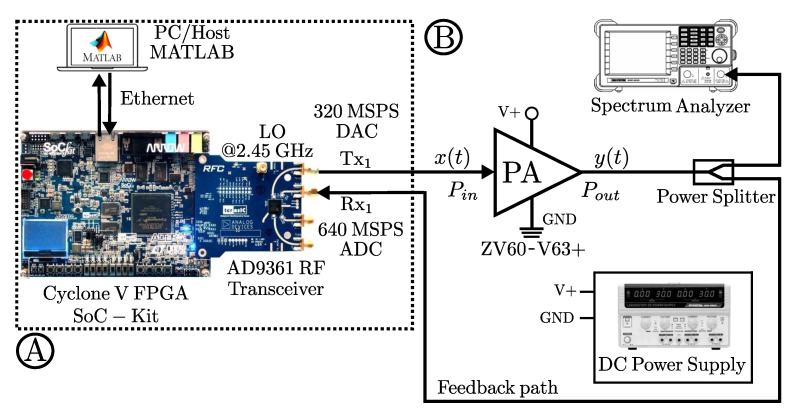
Proposed testbed block diagram of the characterization and linearization for the PA. (**A**) denotes the FPGA digital to RF stage, and (**B**) analog part with devices and measurement equipment.

One can observe from Figure 8 the block diagram of the experimental setup. For the digital part (A) it consists of software and hardware interfacing based on an ADI Linux libiio library runs on a Microblaze ARM (Zynq) architecture that uses an embedded Linux to provide a full-duplex communication from MATLAB and the FPGA hardware implementation. Therefore, the RF transceiver AD9361 transmits the baseband signal; thus, MATLAB casts and stores the data in the DDR memories of the FPGA. The data communication link is made over Ethernet protocol between the ADI Hardware connected to the FPGA-SoC platform. While, in the analog part (B), the RF transceiver generates a stimulus to the ZX60-V63+ PA. The radio-frequency up-conversion path uses a quadrature modulator driven by an internal local oscillator (LO) set at 2.45 GHz frequency to generate upconverted signal.

**Figure 9:**
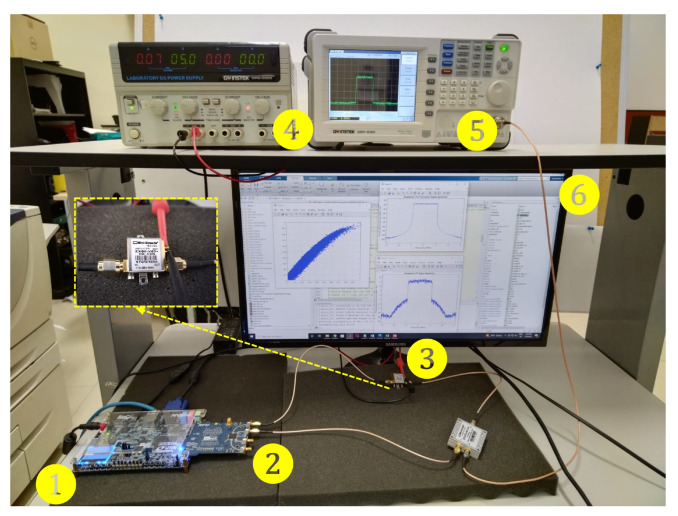
Experimental testbed photograph. Relevant instruments depicted as follows. 1: Altera Cyclone V FPGA SoC-Kit. 2: AD9361 RF Agile Transceiver working at 2.45 GHz center frequency. 3: Power amplifier Mini-Circuits ZX60-V63+. 4: Power Supply GW INSTEK GPS-3303. 5: Spectrum Analyzer GW INSTEK GSP-830. 6: Display HOST PC-MATLAB.

Figure 9 shows a real-time acquisition system; in this process, the DUT is ZX60-V63+ PA for the behavior acquisition. The developed system in the SDR board with a dual channel transceiver operating at 2.45 GHz. The programming of the AD9361 transceiver system provides a wideband capacity ideal for radiofrequency applications; the SDR system has flexibility for mixed-signal baseband selection and internal synthesizers to operate on 2.45 GHz as a carrier frequency. Systems of this type allow using current communication standards, such as frequency-division duplexing (FDD) and interfacing with baseband processors using the 12-bit parallel data port for dual channels. Additionally, an FIR filter is used for internal adjustment of LTE signals with bandwidths of 10 and 15-MHz. The ZX60-V63+ operates at a gain of 18.79 dB for LTE-10 and 18.45 dB for LTE-15, under a bias of 5 V, a 30.72 MSPS is used, with a received signal strength indicator (RSSI) of 103 dB, and an automatic gain control (AGC) to keep amplitude variations and gain for a waveform preamble phase. In this test, the gain control among the blocks achieved by the internal AGC is enabled, such as slow mode gain control. In order to perform the tests power amplifier DUT output measurement is initially measured in the frequency domain with a GW INSTEK GSP-830 as shown in Figure 8. Therefore, the setup for NMSE, PAPR, and ACPR is calculated in MATLAB through the LTE toolbox and the RF Blockset models for Analog Devices RF Transceivers. The data input is cast from the host computer, and the ARRadio card setting tests operates with an AD9361 RF transceiver using a carrier frequency 2.45-GHz and an LTE 10-MHz with a sampling rate of 245.76 MHz and 122.88 for the LTE 15-MHz and performed for an IFFT window size of 1048 points. For the particular validation tests every iteration, the PAPR is obtained. Furthermore, the CCDF is calculated to represent the probability that the envelope actual signal exceeds a defined threshold. The LTE signal data are measured during a given interval to determine the probability under OFDM multiplexing for the PAPR and CCDF are calculated with the real-valued samples data as:

(14) $$\mathrm{PAPR}\left(n\right)=\frac{{\left|x(n)\right|}^{2}}{\frac{1}{N}\sum_{n}^{N-1}{\left|x(n)\right|}^{2}}$$

CCDF is related to the PAPR calculation based on the relation:

(15) $$\mathrm{CCDF}\left(\%\right)=P_{ove}\left(\mathrm{PAPR}>P_{tar}\right)$$

where $P_{ove}$ provides information on the overall time of the signal duration at or above a specific power level, and $P_{tar}$ is denoted as the PAPR target. Previous to this step, the setup system is adequately calibrated so that a synchronized signal is applied properly to the PA input. Please note that we set a calibration power for power peak and average levels at the PA output after each iteration to ensure adequate CFR amplitude levels in the overall system [1]. Figure 9 (1) (2) shows the transceiver set at 2.45 GHz center frequency using the ARRadio card integrates an AD9361 RF transceiver. Once the sample rate has been established, and radio-frequency bandwidth, the signal implemented in MATLAB is designed, as shown in Figure 9 (6), performing with 64-QAM modulation. Moreover, the processes of LTE parameter store can be observed for a specific LTE mode selected, while the transmission and reception path using the RF transceiver board (AD-FMCOMMS3-EBZ) and the MATLAB library to pre- and post-processing captured real-valued data.

## 4. Experimental Design

### 4.1. Pre-Processing and Post-Processing in System Data-Driven Modeling

The procedure for the algorithms extraction is performed to ensure a real-time FPGA implementation. Thus, it is based on the measured outputs which are post-processed in MATLAB with subsampled synchronization to compensate for delays in time and phase; thus, a minimum delay is given by a correlation index (i×5.00ns) according to the clock running at 200 MHz for direct memory access (DMA) between the LPC bus interface of transmitter-receiver or input/output data of the PA using samples of y(n−i) in the AD9361 core for the FPGA data acquisition. In this manner, and as we explained the desirable model for the performance evaluation, considers the model prediction ability that should be tested to verify its robustness compared to CS, RF, and RT models. In the experiments, two cases are considered with datasets of 65,651 and 30,720 samples length, respectively. Both are used to compare the PA model extraction, PD model construction and DPD coefficients estimation to achieve a balance between the modeling and linearization performance, runtime, and computational complexity, which increases with the samples depth. To obtain the PA behavioral models and accuracy for the NMSE of the AM-AM, AM-PM prediction is used to provide the differences between estimated and real-time measurement data, the NMSE is expressed in dB as follows:

(16) $$\mathrm{NMS}E\;{\left(n\right)}_{\mathrm{dB}}=10\log_{10}\left(\frac{\sum_{n=1}^{N}{\left|y\left(n\right)-\bar{y}\left(n\right)\right|}^{2}}{\sum_{n=1}^{N}{\left|y(n)\right|}^{2}}\right)$$

where y(n) is the actual data and $\bar{y}\left(n\right)$ the predicted models in time convergence and accuracy of the estimated PA output.

### 4.2. Framework Development and Application of the PA Models Study Case

**Figure 10:**
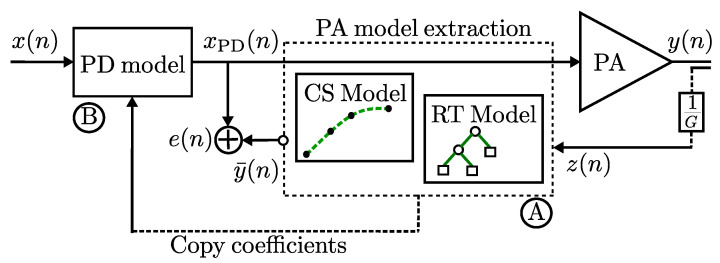
An extract feature block model approach for the PA (connections model referred to a feature surrogate dependent on a complex input envelope with an indirect learning approach). (**A**) denotes the PA model extraction and, (**B**) the PD construction process.

In this work, the performance of LTE signals of 10 and 15-MHz bandwidths are evaluated for commercial LTE applications. For the evaluation a single band LTE signal is used as the input stimulus to drive a wideband power amplifier ZX60-5916MA+ from Mini-Circuits. According Figure 10 a PA model extraction is performed based on the ILA architecture, while procedure (A) can select the parameters for the PA output model y(n) extraction under the spline parameters (i.e., memory *m*, knots, coefficients $c_{i}^{\left(m\right)}$, breakpoints, etc.) and/or RT parameters (i.e., trees, regression trees $\epsilon_{1}$, $\epsilon_{2}$, etc.), prior the algorithm iteration in order to set the training framework adequately for each AM-AM conversion model. Thus, the procedure (B) is copying the coefficients for the PD model construction. The NMSE performance and runtime for the RT model, model extraction, and validation consider a dataset with 30,720 samples acquired from the experimental testbed. RT gives a best performance for ML algorithm in terms in NMSE with a value of −53.0346 dB NMSE in modeling is obtained for the PA RT model output.

### 4.3. Cubic-Spline Algorithm for Coarse-Data Modeling

**Figure 11:**
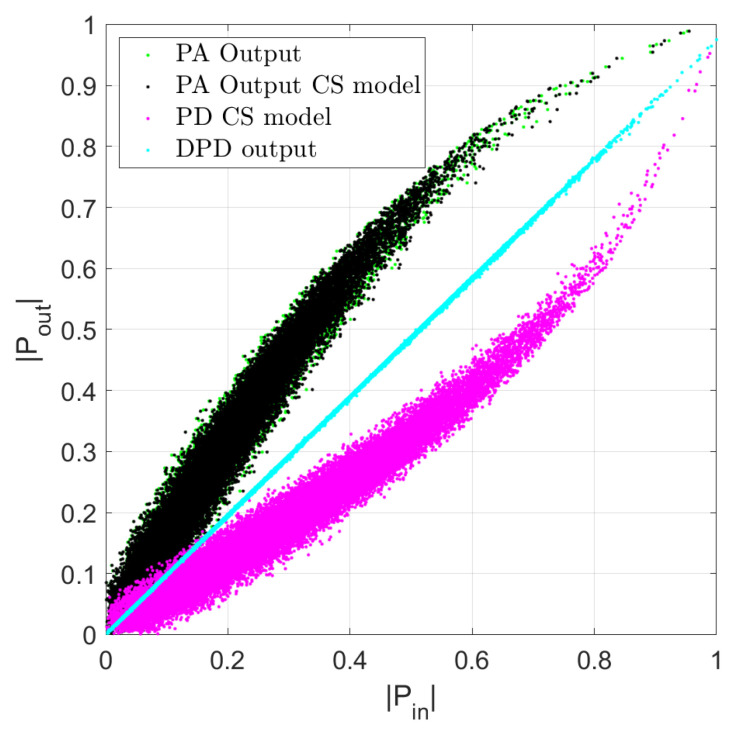
Modeling extraction and evaluation for the LTE 10-MHz and construction process of the AM-AM PA model for the DPD output linearization with CS model basis for a z(n) PD model normalization ≤=0.9999 after 5 iterations reach an NMSE −48.7995 @ 2.45 GHz.

A behavioral model is used in the first case to develop a memoryless predistorer, while the ILA technique is used to correct the residual spectral regrowth ensuring the memory trade-off between complexity and NMSE performance. In this work, they are performed for the LTE digital multiplexing OFDM FDD under 64-QAM modulation. For the residual ACPR spectral regrowth and their CFR metric associated its measured for the transmitter chain distortion, the characteristics can be seen in the AM-AM curve of the DUT PA, modeling, and linearization stage is developed based on the cubic-spline model as shown in Figure 11 and is defined as follows:

(17) $$\begin{array}{cc} y_{\;}^{}\left(n\right) & =\sum_{m=0}^{ML-1}{\;(|x\left(n\;-\;m\right)|}^{2})\;\;x\left(n\;-\;m\right)\;\\ G_{m}\left(x\right) & =\sum_{i=0}^{N_{k}1}c_{i}^{\left(m\right)}\phi_{i}{\left(|x\right|}^{2}) \end{array}$$

Based on the peak gain of the system, the $y_{d}\left(n\right)$ is determined, which is expressed by the following:

(18) $$y\left(n\right)=z\left(n\right)/G_{m}\left(n\right)$$

where $G_{m}\left(n\right)$ is based on the maximum values of x(y) and y(n), as follows

(19) $$G_{m}\left(n\right)=y\left(n\right)\max|x\left(n\right)|/\max|y\left(n\right)|$$

It can be noticed that for even nonlinear behavior effect and parameter estimation can be obtained by different basis functions, such as conventional polynomials, according to Equation (17), assuming a gain factor that can be expanded as in (13) in terms of a one-dimensional CS basis function to the gain $G_{m}{\left(|x\left(n\right)\right|}^{2})$.

### 4.4. Model Complexity Analysis Assessments and Comparison

**Figure 12:**
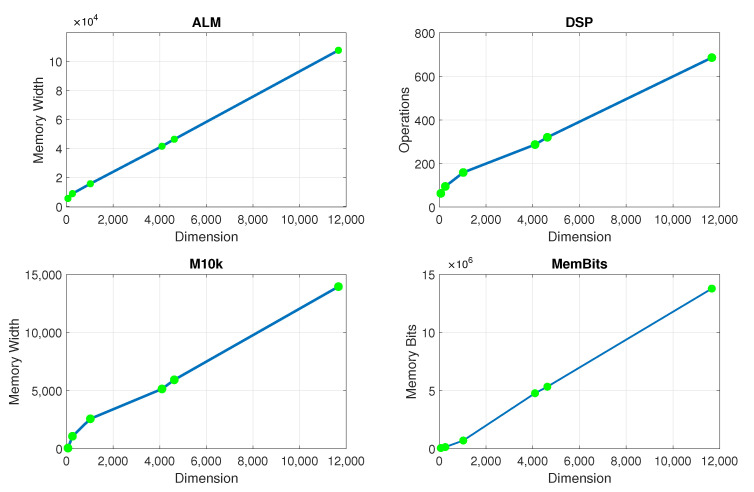
FPGA hardware resource use for the AM-AM PA model with RT implementation (in terms of, ALM, DSP, M10K, and Memory Bits).

The model inversion considers the computation of the 8×8,16×16,32×32,64×64 matrix inversion associated with the hardware resources targeted for each model at the FPGA device Cyclone V SX SoC—5CSXFC6D6F31C6N. For the complexity calculation, the corresponding amount for each matrix dimension considers the following available resources, that is 110K LEs, 41509 ALMs, 5140 M10K memory blocks, which also corresponds with the complexity limits in Figure 12.

It is noted that for simplicity, we assume in this work that the matrix form in Equation (11) is expressed as follows

(20) $$Y=H_{N\times L}C_{L\times 1}$$

where **Y** is the vector of the output signal, C is the coefficient vector, and **H** is the matrix of the model composed of the terms in (13). N is the number of samples. L represents the number of coefficients and L=2K(M+1). After using the LS, the coefficient vector C can be expressed as

(21) $$\tilde{C}={\left(H^{H}H\right)}^{-1}H^{H}Y$$

**Figure 13:**
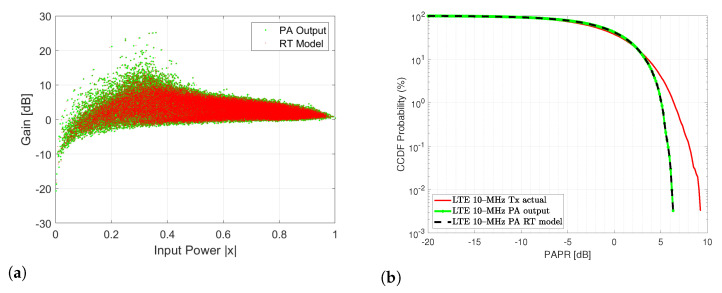
(**a**) Gain for the normalized for output signal using RT prediction model (**b**) CCDF for the PA output signal and the RT prediction model.

As regards the RT model, it is found that the RT prediction closely resembles the PA output in terms of the gain of the normalized PA output signal and the CCDF of the PA output signal. Figure 13 depicts the aforementioned RT modeling performance for the (a) normalized gain (b) CCDF of the PA output signal and the RT model.

**Figure 14:**
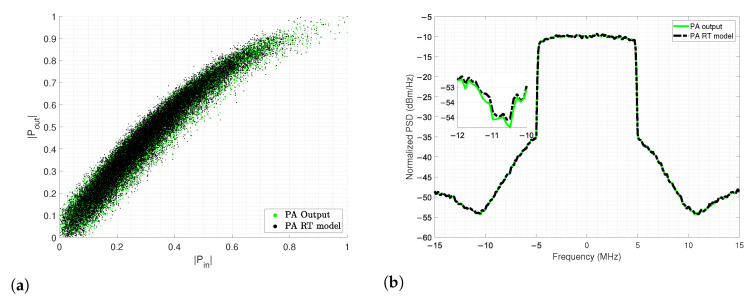
Modeling extraction and evaluation for the; (**a**) Construction process of the AM-AM PA model with RT and (**b**) PSD of the output PA spectral regrowth with RT model basis.

The evaluation of the RT model is performed on the AM-AM characteristic of the PA with noticeable similarity seen in Figure 14a. For further assessments, the normalized power spectrum of the PA output signal is also compared against the RT model prediction in Figure 14b, the model exhibits when a notable gain expansion occur for low power levels, and the corresponding optimum model is desirable for a narrow bandwidth.

**Figure 15:**
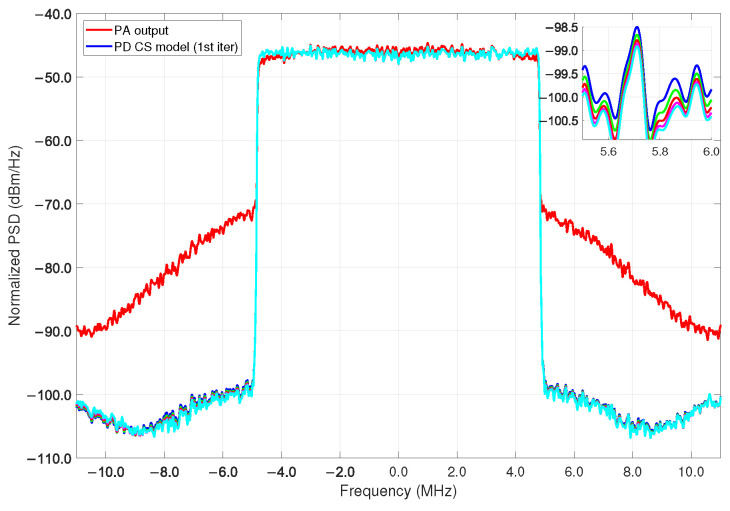
PSD for the LTE 10-MHz PA output signal and the CS prediction model with the actual predistorter z(n) model normalization ≤=0.9999 after 5 iterations reach an NMSE= −48.7995 @ 2.45 GHz.

For the linearization of the power amplifier (CS model) ILA is used; the spectral regrowth of the received signal is observed as a result of the use of the amplifier where DPD is not used as before the PA, while in Figure 15 we observe the spectrum of the received spectra (PA output), while its power values decrease in the bands after parametrization of the CS model (PD CS model) under 5 iterations, that is, required only 90 number of coefficients for the CS model.

The predistorter training is performed in an iterative way; as noted in Figure 15, the algorithm is able to execute a condition to fit the best convergence in terms of NMSE. In this case, the signal is measured in the Tx under a 64-QAM modulation scheme for both bandwidth cases. For the LTE-10 MHz an average output power of −21.11 dBm is obtained with a gain of 18.79 dB while for the LTE-15 MHz a gain of 18.45 dB is achieved. Thus, the validation of the DPD coefficients after training and feeding as the new input to the linearization performance algorithm given its ability to the output signal at the PA then exhibits an adequate spectral regrowth decrease. Figure 15 shows the model iteration of the linearization figures of merit with several selected coefficients for the PA =22 and PD =68 models, respectively, that can obtain the best NMSE with only 90 coefficients used for the cubic-spline algorithm. It is noted that the overall MATLAB procedure acquires data using the PA model extraction, PD model, AM-AM, AM-PM, and linear output model without any iterations.

**Figure 16:**
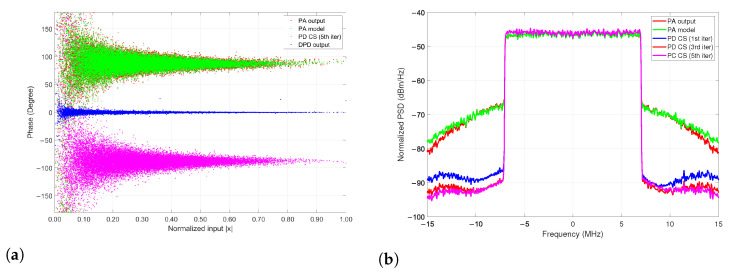
LTE 15-MHz PA output signal: (**a**) Construction process of the AM-PM characteristic model and (**b**) PSD for the LTE 15-MHz PA output signal and the iterative PD CS linearization result.

For these particular validation tests, we iterate the model five times in order to show the runtime convergence for the defined parameters listed in Table 1. For the PA/PD behavioral model extraction using the algorithms of RF, RT, and CS after 5 iterations the results are listed in Table 2. As observed in PSD metrics of Figure 15 and Figure 16b, an effective linearization is achieved after 5 times of the CS algorithm iteration. The validation demonstrates that it is possible to achieve up to 1.52 dB from the first to the fifth iteration for the LTE 10-MHz, and 0.52 dB for the LTE 15-MHz case with a lower runtime, as shown for each bandwidth signal, respectively, before and after the iterations (see CS${}^{†}$ (1st iter) and CS${}^{‡}$ (5th iter) in Table 2).

As proof of the CS modeling capabilities with the LTE 15-MHz bandwidth signal, Figure 16a,b shows the AM-PM characteristic and the PSD, respectively, for both the measured and modeled data from the PA DUT. The ACPR of the PA output and the PD CS model achieves up to 22.57 dB for the best case of −43.90 dBc ACPR reduction. It can be noticed that a linear DPD output model is achievable, and Table 1 collects and summarizes the parameters for the proposed spline-based models employed in the construction of the PA model extraction and PD prediction, respectively.

### 4.5. Discussion

Further studies of state-of-the-art machine learning algorithms applied to PA modeling are compared in Table 3. The present work is devoted to providing a surrogate alternative to models based on RT, RF, and CS algorithms that can be adopted to obtain low complexity PA and PD models for power amplifier characteristics. Additionally, a development SDR platform successfully aids in the search for adequate matrix inversion calculations to validate the proposed models based on an FPGA hardware architecture, whose performance shows an adequate complexity reduction and runtime. Furthermore, we emphasize the high dispersion of AM-AM curves that become high complexity in matrix inversion, but it is demonstrated that the proposed CS and RT can adequately model the PA and PD behavior while having precise NMSE extraction-prediction approximation and learning complexity. The developed models for the PA extraction and update coefficients for the PD model provide a good time convergence and NMSE; this gives an advantage in approximation to very thickness AM-AM and AM-PM behaviors as is in the case of Figure 11 and Figure 16, respectively. With the same principle of obtaining a lower model complexity that yields an effective linearization while using the CS model, we demonstrate the model extraction validation with an affordable runtime when it runs five times iteratively as revealed in Figure 15. The RT model is quite simple yet widely recognized as an effective learning technique for nonlinear problems that establishes the grounds for more elaborated ensemble methods such as RF [26]. These algorithms are able to operate on high-dimensional datasets and select the most biased feature, their comprehensibility nature makes it easier to interpret the model with an enhanced view of performance outcomes [27].

This work expands upon the modeling stage of the PA linearization process and compares the performance of RT, RF, and CS models. It presents an ensemble approach to the RT algorithm that estimates an NMSE term and corrects the model output, this allows the model to better fit the data and achieve greater accuracy at the cost of higher memory consumption. A computational complexity breakdown is realised in order to quantify the performance improvements over previous methods, the analysis is sustained by various measurements on resource use, such as the execution runtime of the algorithm, and the size of the resulting model. An estimation of FPGA memory consumption is calculated for the aforementioned tree-based methods that run on MATLAB, and it is then compared to the CS model with 1 and 5 iterations. The results are presented in terms of NMSE and execution runtime in seconds, while previous works on tree-based models scored an error of −30.93 dB with an execution runtime of 16.82 seconds [20], the improved approach is able to obtain an error of −53.03 dB in 2.41 seconds in the case of the RT algorithm. The addition of an error term, and optimizations to parameters and memory consumption contribute to an overall enhancement of the model. The strong precision on RT is corroborated by the NMSE results of Table 2, having a sufficient runtime compared to other approaches. The RF method introduces different models with more diverse predictions that, when combined reduces variance on the model which in turn leads to a more general output.

## 5. Conclusions and Future Work

In this work, a feature comparison for algorithm performance is developed to provide a fair comparison of ML methods including RT, RF, and a cubic-spline basis for PA modeling. The proposed validation is carried out with 64-QAM modulation LTE-10 and 15-MHz signal bandwidths to develop the resulting algorithm to exploit feature characteristics of DPD solution as surrogate models in terms of extraction and evaluation, while also accurate enough to enhance convergence and steer the iterations target to the global optimum following the same approach by using the AM-AM/AM-PM real-valued data characteristics. The measurement examples for DUT performance based on the ILA architecture offer real-time convergence on results for each algorithm developed in order to obtain a precise approximation for the PA (e.g., extraction, prediction, linearized model) under test while with LTE signals are used as stimulus. Finally, for the data conditioning aspects from three models compared the NMSE is provided with a good accuracy when the complexity is preserved according to the smoothness-straightness dynamic dependencies for the AM-AM characteristics considered at each developed algorithm while preserving model with a few samples, such as the ML based and feature basis splines construction to give a fair comparison. Further development of the present models considers an engine based on the FPGA-based system present in order to adequate precision in terms of floating point.

## Figures and Tables

**Table 1:** Parameters summary for the PA extraction and PD prediction with CS model.

Signal	Parameters		Coefficients
	**Memory PA**	**Memory PD**	
LTE 10-MHz	2	5	90
LTE 15-MHz	3	6	183

**Table 2:** NMSE and runtime convergence for the LTE 10-MHz and LTE 15-MHz signals.

Model	LTE 10-MHz	LTE 10-MHz		LTE 15-MHz	LTE 15-MHz	
	NMSE (dB)	Runtime (*s*)		NMSE (dB)	Runtime (*s*)	
		**Training**	**Prediction**		**Training**	**Prediction**
Random Forest	−25.0470	10.7984	0.631920	−26.1236	12.5964	0.76038
Regression Tree	−53.0346	2.37000	0.048308	−53.7618	2.84020	0.15436
CS${}^{†}$ (1st iter)	−47.2767	0.1925		−41.6136	0.3586	
CS${}^{‡}$ (5th iter)	−48.7995	1.16		−42.1412	1.8889	

CS^†^ and CS^‡^ denote the prediction results shown in Figure 15 and Figure 16a,b.

**Table 3:** Performance comparison with some Machine Learning-based algorithms.

Model	Freq (MHz)	Signal/BW (MHz)	NMSE (dB)	PAPR (dB)	ACPR (dBc)	Ref.
Regression Tree *	2450	LTE/10 & 15-MHz	−53.03 *	9.3	−43.5	This Work
Random Forest	3600	LTE/30-MHz	−48.40	9.6	−49,−56	[11]
Gradient Boosting	1900	5G NR/60-MHz	−38.00	–	−43.46	[28]
Neural Network	1900	5G NR/60-MHz	−31.58	–	−39.97	[28]
Chebyshev Polynomial LSTM ${}^{†}$	2800	5G NR/100-MHz	−36.12	9.6	–	[29]
AVDTDNN	3750	OFDM/40-MHz	−38.50	6.9	−48.95/ −49.09	[30]

* After PA model extraction for the proposed RT model without any iteration. † A long short-term memory (LSTM) network is considered.

## Data Availability

Not applicable.
